# Recovery of Severe Acute Kidney Injury in a Patient with COVID-19: Role of Lung Ultrasonography

**DOI:** 10.24908/pocus.v7iKidney.15343

**Published:** 2022-02-01

**Authors:** Varun Madireddy, Daniel W Ross, Deepa A Malieckal, Shamir Hasan, Azzour Hazzan, Hitesh H Shah

**Affiliations:** 1 Division of Kidney Diseases and Hypertension, Donald and Barbara Zucker School of Medicine at Hofstra/Northwell Great Neck, NY USA

**Keywords:** AKI, COVID-19, lung ultrasound, point of care ultrasound

## Abstract

Acute kidney injury (AKI) is recognized as a complication of COVID-19 among hospitalized patients. Lung ultrasonography (LUS) can be a useful tool in the management of COVID-19 pneumonia when interpreted correctly. However, the role of LUS in management of severe AKI in the setting of COVID-19 remains to be defined. We report a 61-year-old male who was hospitalized with acute respiratory failure from COVID-19 pneumonia. In addition to requiring invasive mechanical ventilation, our patient developed AKI and severe hyperkalemia requiring urgent dialytic therapy during his hospital stay. Our patient remained dialysis dependent despite subsequent recovery of lung function. Three days following discontinuation of mechanical ventilation, our patient developed a hypotensive episode during his maintenance hemodialysis treatment. A point of care LUS performed soon after the intradialytic hypotensive episode found no extravascular lung water. Hemodialysis was discontinued and the patient was initiated on intravenous fluids for one week. AKI subsequently resolved. We consider LUS an important tool in identifying COVID-19 patients that would benefit from intravenous fluids following recovery of lung function.

## Introduction

COVID-19, the disease caused by SARS-CoV-2, is commonly associated with acute respiratory failure among hospitalized patients 1. Acute kidney injury (AKI) has also been reported in a significant percentage of hospitalized patients with COVID-19, many of whom have required dialysis. A restrictive fluid balance in patients with acute lung injury is associated with fewer days of invasive mechanical ventilation 2. As a result, many physicians employ a strategy of keeping lungs “dry” through diuretics, and when necessary, ultrafiltration. 

Lung ultrasonography (LUS) is a useful tool for evaluating pulmonary disease 3, 4. A recent Italian study reviewed the importance of LUS in management of patients with COVID-19 pneumonia 5. Here, we describe a case wherein LUS provided critical information to successfully treat AKI in a patient recovering from COVID-19 pneumonia.

## Case Presentation 

A 61-year-old male with no significant past medical history was hospitalized with COVID-19 at our tertiary care facility in Queens, New York in early April 2020. The patient presented with two weeks of intermittent fevers at home and was tested positive for COVID-19 prior to hospitalization. On presentation, the patient’s body temperature was 103°F, blood pressure (BP) was elevated at 184/90 mmHg, heart rate was 110 bpm, body weight was 100 kg, and pulse oxygen saturation was significantly low at 74% on non-rebreather O2 mask. Blood work showed blood urea nitrogen of 15 mg/dL and serum creatinine of 0.97 mg/dL. Arterial blood gas was significant for pH 7.13, PaCO_2 _80 mmHg, measured HCO_3_ 23 mmol/L. Chest x-ray showed bilateral opacities. The patient was emergently intubated and placed on mechanical ventilation on hospital day 1.

On hospital day 2, the patient was noted to have AKI with severe hyperkalemia and persistent acidosis. Blood urea nitrogen was 24 mg/dL, serum creatinine increased to 1.49 mg/dL, serum potassium was significantly elevated at 7.9 mEq/L, and pH remained low at 7.1. Nephrology was consulted and continuous veno-venous hemodiafiltration (CVVHDF) was urgently initiated. On hospital day 7, the patient was transitioned from CVVHDF to intermittent hemodialysis (IHD). The patient was oliguric from hospital day 2 to 11. He was successfully extubated on hospital day 14. On hospital day 17, blood urea nitrogen and serum creatinine remained significantly elevated at 198 mg/dL and 7.88 mg/dL, respectively. On the same day, the patient became hypotensive during his IHD treatment. At the start of IHD treatment, the patient’s BP was 146/94 mmHg and after 50 minutes of dialysis, BP decreased to 76/59 mmHg. He was not requiring supplemental oxygen at that time but was subjectively dyspneic and tachypneic to 30 breaths per minute.

**Figure 1:**
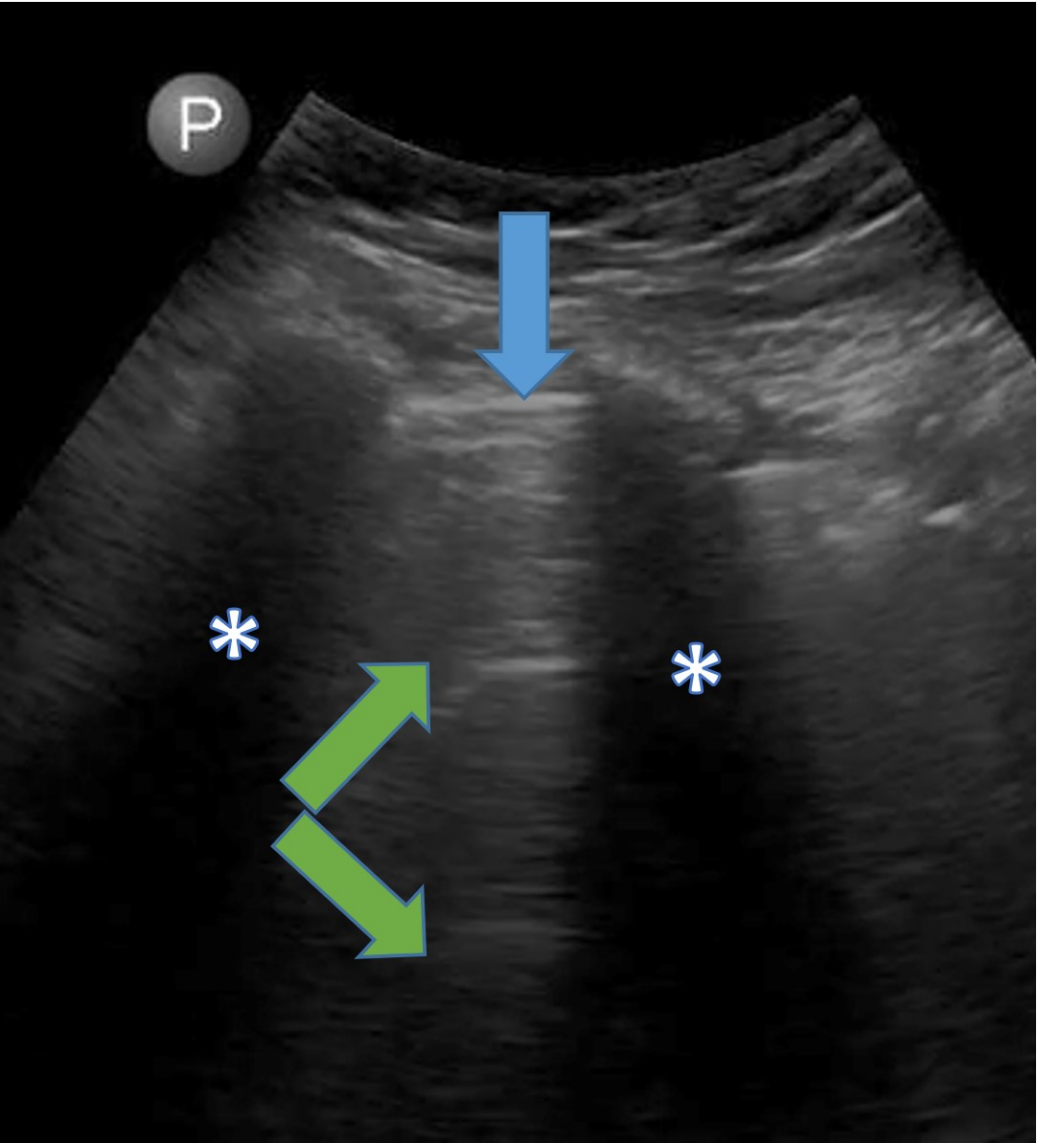
Lung ultrasound image obtained using a curvilinear probe. Blue arrow shows the pleural line,Green arrows show A-lines. Asterisks indicate rib shadows.

An 8 zone lung ultrasound was performed at bedside soon after the hypotensive episode. Findings demonstrated predominantly A-lines with intact lung sliding (Figure 1). The patient’s obese body habitus and the availability of only a curvilinear ultrasound probe precluded us from performing a point of care echocardiogram. Following LUS the patient was initiated on intravenous saline infusion and further dialytic therapy was discontinued. After seven days of continous saline, blood urea nitrogen improved to 56 mg/dL and serum creatinine decreased to 1.99 mg/dL. After a month of hospitalization, serum creatinine further improved to 1.00 mg/dL at discharge.

## Discussion

Our patient had acute respiratory distress syndrome (ARDS) from COVID-19 pneumonia. Hospital course was complicated by oliguric AKI, severe hyperkalemia, and acidosis requiring initiation of dialytic therapy. During the initial part of his hospitalization, dialytic therapy played a key role in oxygenating and ventilating our patient. Our patient remained dialysis dependent despite resolving respiratory failure. Three days following extubation, the patient developed an episode of intradialytic hypotension. LUS was performed for realtime evaluation of his lung disease. His lung ultrasound findings revealed a normal aeration pattern suggesting low level of extravascular lung water and possibly a pre-renal state. 

Air is a poor conductor of sound. When an ultrasound wave passes from the pleural surface to the underlying air the mismatch in acoustic impedance sends a strong echo back to the interface between the skin and the probe. When the echo meets this second strong reflector (the skin-probe) it is reverberated back again to the pleural line. This reverberation is misinterpreted by the ultrasound software and appears as a linear structure deep to the pleural line. These artifacts are known as A-lines and are consistent with a normal aeration pattern. In their seminal work, Lichtenstein et al. have demonstrated that A-lines are strongly predictive of a low pulmonary capillary wedge pressure (PCWP) 4. When an ultrasound wave meets a water-thickened interlobular septa in the lung, a B-line reverberation artifact is seen. This appears as a straight line that is perpendicular to the pleura and moves as the lung slides. B-line pattern is when three or more B lines are seen at two rib interspaces, bilaterally. B-line pattern can indicate cardiogenic pulmonary edema when the B lines are associated with a smooth pleural line and are distributed homogenously in an interspace. B-lines can also be seen in COVID-19 pneumonia and in the resultant ARDS but typically have an irregular or fractured pleura with sub-pleural consolidations. These findings typically resolve to A-lines with clinical improvement 6. 

Our patient was obese and recovering from COVID-19 pneumonia with ARDS. He was dyspneic and tachypneic. His volume status was very difficult to ascertain due to his body habitus. We were concerned that excessive fluid resuscitation could worsen his respiratory status if he was still exhibiting signs of ARDS. In our patient, the presence of A-lines and absence of B-line pattern argued in favor of a resolved COVID-19 pneumonia and ARDS and in favor of a normal or low PCWP. These findings gave us the reassurance to attempt a trial of fluid hydration. 

Lung ultrasonography should not be performed in isolation. Ideally, a point of care echocardiogram would have been done as well. Unfortunately, we only had a curvilinear probe at our disposal at the time. Curvilinear probes can be used to image the inferior vena cava (IVC). The patient’s body habitus prevented an adequate window to view the IVC. Intradialytic hypotension can also be caused by pericardial effusion and pulmonary embolism. A limited echo in this case would have been valuable to look for effusion, right ventricular strain, and cardiac contractility. Of note, a referral echocardiogram with contrast was done a few days later. The study was technically limited but ruled out pericardial effusion. 

A restrictive fluid approach is commonly used in patients with acute lung injury. This strategy, while crucial for helping with oxygenation, can also lead to pre-renal AKI. Lung ultrasonography is a reliable tool to get real-time, point of care information about a patient’s pulmonary status. Based on our experince, LUS may be an important tool in identifying COVID-19 patients that would benefit from intravenous fluids following recovery of lung function.

## Conflicts of Interest and Funding

The authors have no conflicts of interest or funding to report.

## Informed Consent 

The Northwell Health Institutional Review Board approved this case as minimal-risk research using data collected for routine clinical practice and waived the requirement for informed consent however verbal consent was obtained from the next of kin.
